# Identification and Validation of a Prognostic Risk-Scoring Model Based on Ferroptosis-Associated Cluster in Acute Myeloid Leukemia

**DOI:** 10.3389/fcell.2021.800267

**Published:** 2022-01-21

**Authors:** Jinghua Wang, Zewei Zhuo, Yanjun Wang, Shuo Yang, Jierong Chen, Yulian Wang, Suxia Geng, Minming Li, Xin Du, Peilong Lai, Jianyu Weng

**Affiliations:** ^1^ Department of Hematology, Guangdong Provincial People’s Hospital, Guangdong Academy of Medical Sciences, Guangzhou, China; ^2^ Department of Gastroenterology, Guangdong Provincial People’s Hospital, Guangdong Academy of Medical Sciences, Guangzhou, China; ^3^ Department of Urology, Sun Yat-sen University Cancer Center, State Key Laboratory of Oncology in South China, Collaborative Innovation Cencer for Cancer Medicine, Guangzhou, China; ^4^ Department of Cardiovascular Division, Peking University Shenzhen Hospital, Shenzhen, China; ^5^ Department of Laboratory Medicine, Guangdong Provincial People’s Hospital, Guangdong Academy of Medical Sciences, Guangzhou, China

**Keywords:** ferroptosis, acute myeloid leukemia, prognosis, nomogram, immune infiltration, response to chemotherapy

## Abstract

**Background:** Emerging evidence has proven that ferroptosis plays an important role in the development of acute myeloid leukemia (AML), whereas the exact role of ferroptosis-associated genes in AML patients’ prognosis remained unclear.

**Materials and Methods:** Gene expression profiles and corresponding clinical information of AML cases were obtained from the TCGA (TCGA-LAML), GEO (GSE71014), and TARGET databases (TARGET-AML). Patients in the TCGA cohort were well-grouped into two clusters based on ferroptosis-related genes, and differentially expressed genes were screened between the two clusters. Univariate Cox and LASSO regression analyses were applied to select prognosis-related genes for the construction of a prognostic risk-scoring model. Survival analysis was analyzed by Kaplan–Meier and receiver operator characteristic curves. Furthermore, we explored the correlation of the prognostic risk-scoring model with immune infiltration and chemotherapy response. Risk gene expression level was detected by quantitative reverse transcription polymerase chain reaction.

**Results:** Eighteen signature genes, including ZSCAN4, ASTN1, CCL23, DLL3, EFNB3, FAM155B, FOXL1, HMX2, HRASLS, LGALS1, LHX6, MXRA5, PCDHB12, PRINS, TMEM56, TWIST1, ZFPM2, and ZNF560, were developed to construct a prognostic risk-scoring model. AML patients could be grouped into high- and low-risk groups, and low-risk patients showed better survival than high-risk patients. Area under the curve values of 1, 3, and 5 years were 0.81, 0.827, and 0.786 in the training set, respectively, indicating a good predictive efficacy. In addition, age and risk score were the independent prognostic factors after univariate and multivariate Cox regression analyses. A nomogram containing clinical factors and prognostic risk-scoring model was constructed to better estimate individual survival. Further analyses demonstrated that risk score was associated with the immune infiltration and response to chemotherapy. Our experiment data revealed that LGALS1 and TMEM56 showed notably decreased expression in AML samples than that of the normal samples.

**Conclusion:** Our study shows that the prognostic risk-scoring model and key risk gene may provide potential prognostic biomarkers and therapeutic option for AML patients.

## Introduction

Acute myeloid leukemia (AML) is an aggressive malignant neoplasm arising within bone marrow, characterized by aberrant accumulation of myeloid precursors (Lim et al., 2017). As a deadliest form of acute leukemia, patients with AML have a dismal 5-year survival rate of 28.3%, and most cases still relapse frequently after remission, leading to a poor prognosis (Lewis et al., 2021; Newell and Cook, 2021). Recently, it is demonstrated that the molecular genetic abnormalities are significantly associated with prognosis in AML, which can serve as a comprehensive risk-stratification system and an effective therapy option (Bernard et al., 2020; Dohner et al., 2020). Despite the advances in exploring the prognostic markers for AML patients, patients belonging to the same group may also show different prognosis due to their clinical process variability (Docking et al., 2021). Therefore, a novel prognostic risk-scoring model is urgently required to improve the current risk stratification and provide more therapeutic options, which eventually improve AML patients’ outcomes.

Ferroptosis is a crucial, iron-dependent regulated cell death driven by excessive accumulation of lipid hydroperoxides (Zou et al., 2020). During the process of ferroptosis, lipid metabolism is altered with the concomitant elevation and massive accumulation of lipid-based reactive oxygen species (ROS) levels in cells, leading to cell damage or even death (Cui et al., 2014). Previous studies indicate that ferroptosis is widely implicated in cancer development and therapy resistance, including particularly the increased sensitivity of AML cells to chemotherapeutic drugs (Yu et al., 2015; Chen et al., 2021; Mao et al., 2021). Several of the ferroptosis-related genes have also been proven to play a vital role in AML (Grignano et al., 2020; Birsen et al., 2021). In addition, the effect of immune infiltration on the ferroptosis and the prognosis of AML becomes more and more significant as well. For instance, CD8^+^ T cells could effectively restore T cell function and improve their antitumor activity by inhibiting ferroptosis (Ma et al., 2021). Moreover, there is emerging evidence that a high proportion of natural killer (NK) cells is associated with death before remission (Park et al., 2018). However, the degree to which ferroptosis influences survival and treatment strategy of AML remains unknown.

Considering the significant value of ferroptosis in AML, we identify differentially expressed genes that are affected by ferroptosis status and then construct a prognostic risk-scoring model and effective nomogram. Furthermore, we explore the relationship of the risk model with immune infiltration and response to chemotherapy, which extends its clinical value for AML patients’ prognosis. We also evaluate the expression of several risk genes in primary AML samples.

## Materials and Methods

### Data Collection

RNA sequencing (RNA-seq) data and corresponding clinical data of 151 AML patients were obtained from the TCGA database (https://portal.gdc.cancer.gov), and these were selected as the training set. The GSE71014 data set included 104 AML samples that were downloaded from the GEO database (https://www.ncbi.nlm.nih.gov/gds/), which were used as the validation set. Another validation set included 155 samples obtained from the TARGET database (https://ocg.cancer.gov/programs/target). “Limma” R package was used to normalized the gene expression profiles. In addition, 261 ferroptosis-related genes (FRGs) were collected from ferroptosis-associated gene sets from FerrDb (http://www.zhounan.org/ferrdb) and the previous literature (Liang et al., 2020; Zhuo et al., 2020).

### Consensus Clustering Analysis Based on Ferroptosis-Related Genes

AML patients from the TCGA database were grouped into different groups based on 261 FRGs by using the “ConsensusClusterPlus” R package, and then Kaplan–Meier (KM) overall survival curves between different clusters were performed by the “survival” R package. Principal component analysis (PCA) was applied to assess sample clustering. “DESeq2” R package was used to screen differentially expressed genes (DEGs) between different clusters (|logFC| > 1.5, FDR < 0.05). Gene Ontology (GO) enrichment analysis and Kyoto Encyclopedia of Genes and Genomes (KEGG) analysis were performed to select and visualize significant enriched ferroptosis-associated GO terms and KEGG pathways in DEGs.

### Construction and Validation of the Prognostic Risk-Scoring Model Based on Ferroptosis-Related Clusters

To select prognosis-related genes (*p* < 0.05), we performed a univariate Cox regression analysis in these DEGs. Gene expression and the KM curve of the six most significant genes among them was presented as well. LASSO regression was then applied to remove redundant prognostic genes for developing the prognostic model. Eighteen genes were ultimately retained, and a risk score was calculated according to the following formula:

$$\mathrm{Risk}\;\mathrm{score}=\sum_{i=1}^{n}\left(Coef_{i}\times x_{i}\right),$$

where $Coef_{i}$ is coefficient and $x_{i}$ is the *z*-score-transformed relative expression value of each selected gene.

After risk model construction, the AML samples from the TCGA cohort were categorized into high- and low-risk groups. The survival difference of both groups was compared using the “survival” and “survminer” R packages, and 1-, 3-, and 5-year receiver operator characteristic (ROC) curve analyses were performed by “time-ROC” R packages. Other AML cohorts from the GEO (GSE71014) and TARGET databases (TARGET-AML) were applied for validation, and the risk score calculation, risk subgroups, survival analysis, and ROC curves were conducted in the same way.

### Prognostic Analysis of Prognostic Risk-Scoring Model

To further explore the relationship between clinicopathologic characteristics and AML patients’ prognosis, we extracted the clinical data from the TCGA cohort, and these variables included AML risk category, age, gender, race, class, and risk score. Univariate and multivariate Cox regression analyses were performed to identify the independent prognostic factors.

### Establishment of the Predictive Nomogram

A nomogram was constructed to visualize the relationship between variables and the prognostic model by the “rms” R package. 3-years and 5-years calibration curves were applied to discriminate and predict the values of a nomogram. To better illustrate the role of our risk score in AML development, we analyzed the relationship between our risk score and different clinical features (AML risk category, age, class, and status).

### Immune Infiltrates Correlation of Prognostic Model

Immune infiltration analysis by the CIBERSORT algorithm was used to evaluate different types of immune cell expression between high- and low-risk gene expression groups. The linear correlation of risk score and immune cell components (T cells CD4 naïve, monocytes, macrophage M2, mast cells resting) was analyzed by the R package “ggstatsplot.” The multigene correlation map was displayed by the R package “pheatmap.”

### Mutation Distribution of Prognostic Model

Mutation data of AML was obtained from the TCGA database and somatic mutations between the high- and low-risk groups were visualized using R package “maftools.”

### Chemotherapeutic Response Prediction of the Prognostic Model

Due to missing drug data in the TCGA-LAML data set, we used an immunotherapeutic data set of bladder cancer (IMvigor210 cohort) to predict the chemotherapeutic response of our prognostic model. Risk score distribution of patients with different drug response groups were used to validate the efficiency of a prognostic model.

### Primary AML Sample Collection and Quantitative Reverse Transcription Polymerase Chain Reaction Analysis

Bone marrow or peripheral blood samples were donated by patients with primary AML in Guangdong Provincial People’s Hospital, and their medical information was collected with informed patient consent and in accordance with the Declaration of Helsinki. This study was approved by the Ethics Committee of the Guangdong Provincial People’s Hospital. Diagnosis of patients was based on morphology using the French-American-British (FAB) classification, immunophenotyping, cytogenetics, and molecular genetics. The complete response (CR) was defined as BM blasts <5%; absence of circulating blasts and blasts with Auer rods; absence of extramedullary leukemia; absolute neutrophil count >1.0 × 10^9^/L; platelet count >100 × 10^9^/L. Total RNA was extracted by an E. Z. N. A. Total RNA Isolation Kit (Omega, GA, United States). The generation of cDNAs from reverse transcription was performed by PrimeScript™ RT-PCR kit (TaKaRa, Otsu, Japan). According to the manufacturer instructions of Biorad CFX Connect (Bio-Rad Laboratories, CA, United States), we conducted the qRT-PCR by using SYBR Premix Ex Taq (TaKaRa, Otsu, Japan). The specific operation steps of qRT-PCR were performed as described previously (Tu et al., 2020). ABL was used as an internal control gene. The primers of LGALS1, ZFPM2, and TMEM56 are as follows: LGALS1 forward (5′-GCA​CTT​CAA​CCC​TCG​CTT​CA-3′), reverse (5′-TCC​TTG​CTG​TTG​CAC​ACG​AT-3′); ZFPM2 forward (5′-GCC​GGC​ACG​AAA​CAT​ACA​T-3′), reverse (5′-GGC​AGG​CAC​TTT​GTT​GGA​A-3′); TMEM56 forward (5′-GCT​GGC​ATA​CAT​TGG​GAA​TTT​T-3′), reverse (5′-CTT​CAA​AGA​ACC​ACC​GCT​GAT​T-3′).

### Statistical Analysis

R software was used to analyze all statistics, and *p* ≤ 0.05 was considered statistically significant. Unless otherwise indicated, Student’s *t*-test was used to test for statistical comparisons.

## Results

### Classification of AML Based on the Ferroptosis-Related Gene Sets

**FIGURE 1:**
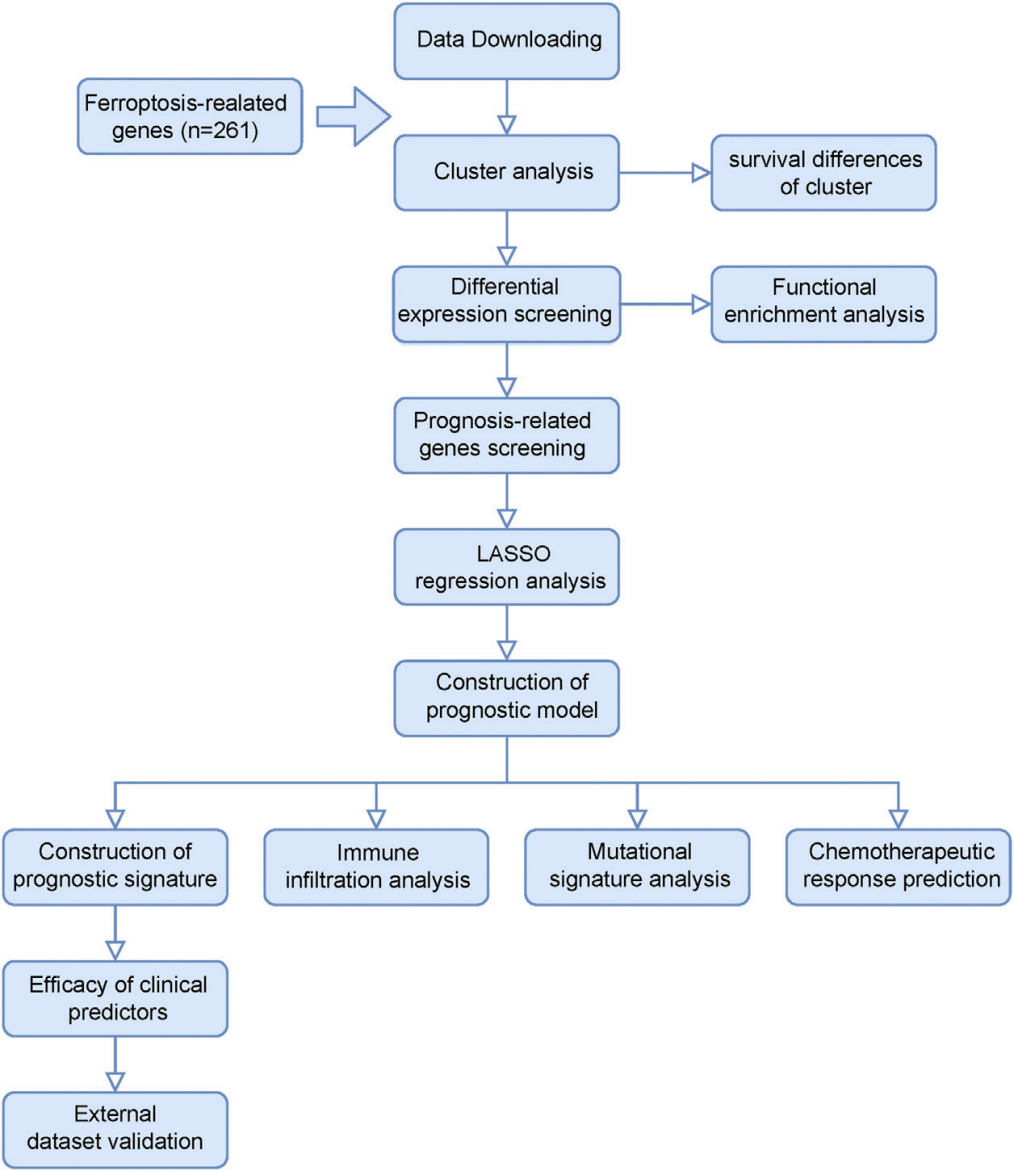
Flow chart of our study design.

**FIGURE 2:**
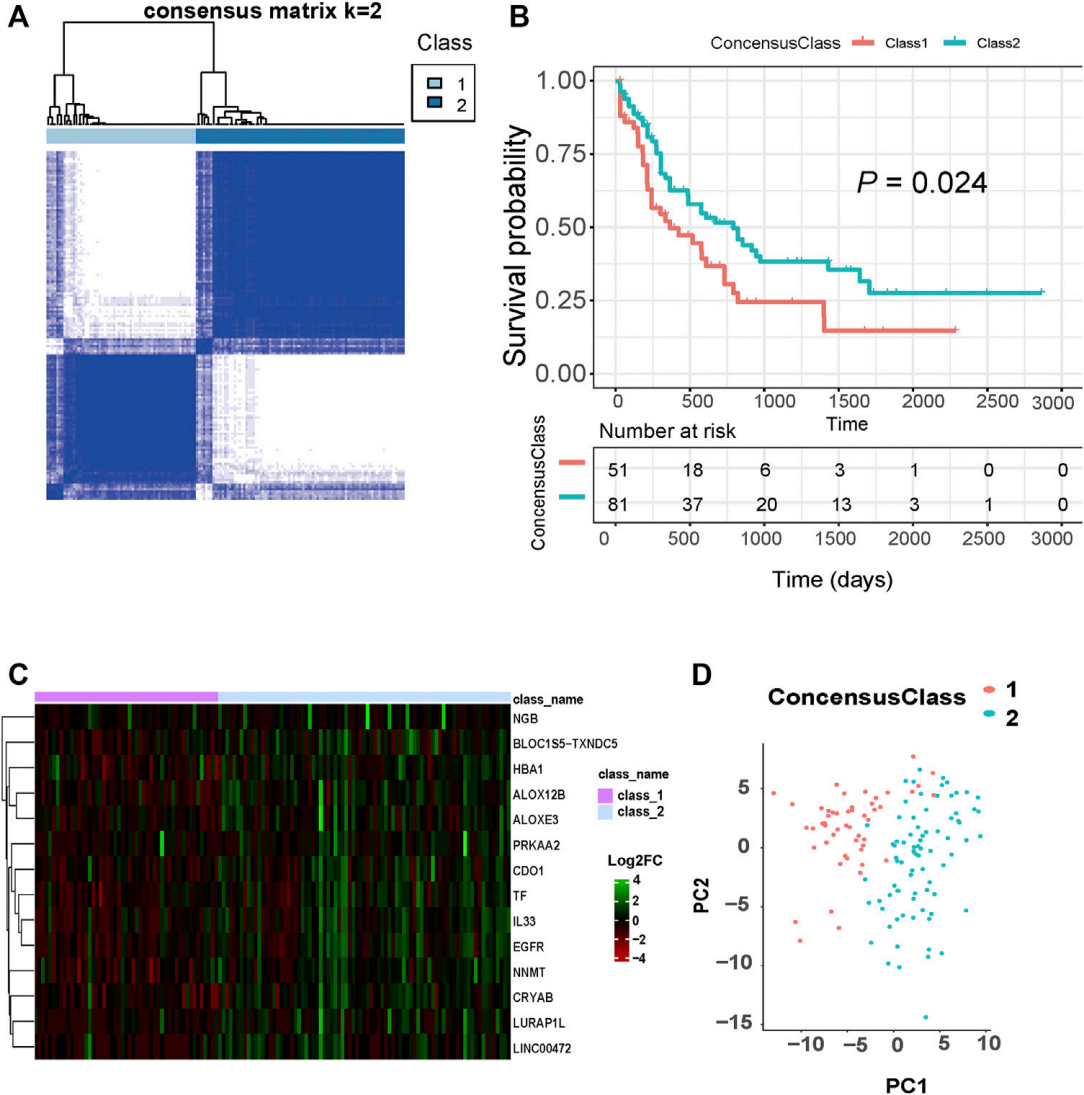
Classification of AML based on ferroptosis-related genes. **(A)** The optimal AML classification according to consensus matrix (k = 2). **(B)** KM survival analysis for the two subtypes. **(C)** Heat maps of these significantly differential ferroptosis-related genes between two subtypes. **(D)** PCA analysis of sample distributions based on ferroptosis-affected genes.

Figure 1 shows the flow chart of our study design. We extracted AML patients’ RNA-seq data and corresponding clinical information from the TCGA database (*n* = 151). Based on 261 FRGs, we conducted a consensus clustering analysis with all AML samples. When consensus matrix K = 2, AML samples can be well grouped into classes 1 and 2 (Figure 2A). KM survival curves of two clusters indicate that class 2 AML patients had better survival compared with those in class 1 (log-rank, *p* = 0.024, Figure 2B). Figure 2C presented a heat map of significantly different ferroptosis-related gene expressions in two clusters. PCA analysis of sample distributions based on ferroptosis-affected genes show a good clustering quality (Figure 2D).

### Functional Enrichment Analysis of DEGs Between Subgroups

A total of 1894 DEGs were identified between the two clusters [*p* < 0.05 and absolute (log2 fold change) > 1.5]. We performed GO enrichment and KEGG pathway analysis for these DEGs, and enriched pathways containing FRGs were selected to draw the Sankey diagrams. In the molecular function subontology, GO terms related to tetrapyrrole binding (GO:0046906), pattern recognition receptor activity (GO:0038187), receptor ligand activity (GO:0048018), heme binding (GO:0020037), and amyloid-beta binding (GO:0001540) were significantly enriched (Supplementary Figure S1A). Under the biological process, ROS metabolic process (GO:0072593), cellular response to external stimulus (GO:0071496), response to drug (GO:0042493), response to extracellular stimulus (GO:0009991), and response to nutrient levels (GO:0031667) were significantly enriched (Supplementary Figure S1B). The cellular component enrichment revealed that DEGs containing FRGs mainly involve endocytic vesicle (GO:0030139), vesicle lumen (GO:0031983), external side of plasma membrane (GO:0009897), neuronal cell body (GO:0043025), and early endosome (GO:0005769) (Supplementary Figure S1C). The KEGG pathway analysis indicates that these DEGs are mainly enriched in legionellosis, neutrophil extracellular trap formation, viral protein interaction with cytokine, and others (Supplementary Figure S1D). The detailed information is provided in Supplementary Tables S1–S4.

### Identification and Validation of Prognostic Model Based on Ferroptosis-Related Clusters

**FIGURE 3:**
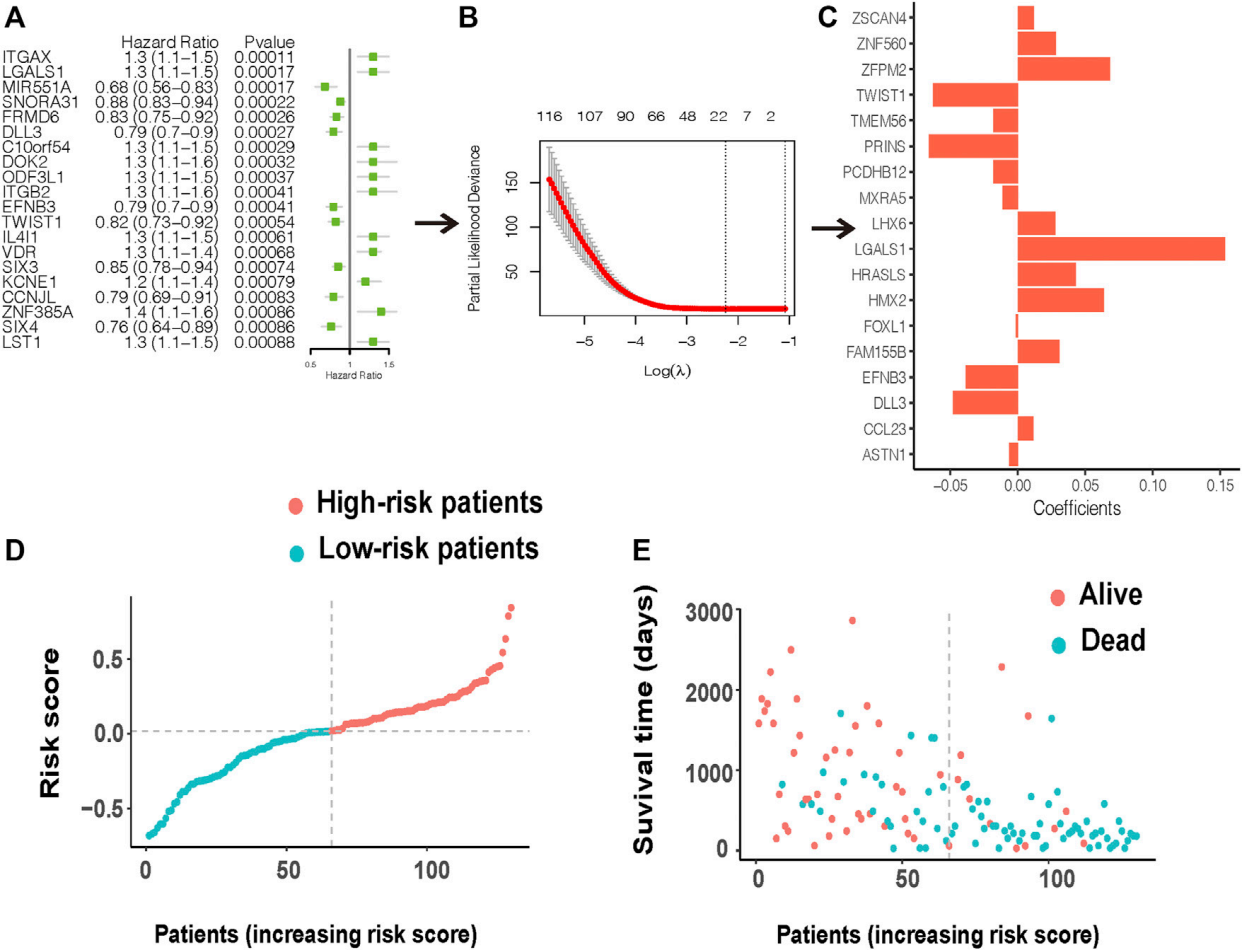
Construction of a prognostic risk model. **(A)** A forest plot of the top 20 prognosis-related DEGs. **(B)** LASSO variable screening process. **(C)** 18 prognostic genes were eventually selected after LASSO regression to establish a prognostic model. **(D)** Distribution of the risk score for the AML patients. **(E)** Patients’ status based on the risk score.

By performing univariate Cox regression analysis, 287 DEGs were significantly associated with patient survival (*p* < 0.05). Integrin subunit alpha X (ITGAX), galectin 1 (LGALS1), and microRNA 551a (MIR551A) were the top 3 prognosis-related DEGs with the smallest *p*-values. The expression of ITGAX and LGALS1 were highly expressed in class 1 compared with class 2, whereas the high expression of ITGAX and LGALS1 had worse survival (Supplementary Figures S2A,B). In opposition, MIR551A was more highly expressed in class 2 than class 1, and high expression of MIR551A had better survival (Supplementary Figure S2C). Figure 3A presents the forest plot of the top 20 prognosis-related DEGs with the smallest *p*-values (the full information is shown in Supplementary Table S5). To further construct an effective prognostic risk-scoring model, we used Lasso regression to narrow down the range of candidate genes (Figure 3B). Eighteen genes [zinc finger protein 560 (ZNF560), zinc finger and SCAN domain containing 4 (ZSCAN4), LIM homeobox 6 (LHX6), twist family bHLH transcription factor 1 (TWIST1), Lower forkhead box L1 (FOXL1), zinc finger protein, FOG family member 2 (ZFPM2), H6 family homeobox 2 (HMX2), Astrotactin-1 (ASTN1), delta-like protein 3 (DLL3), protocadherin beta 12 (PCDHB12), psoriasis-associated nonprotein coding RNA induced by stress (PRINS), TLC domain containing 4 (TMEM56), HRAS like suppressor (HRASLS), family with sequence similarity 155 member B (FAM155B), C-C motif chemokine ligand 23 (CCL23), galectin 1 (LGALS1), ephrin B3 (EFNB3), and matrix remodeling associated 5 (MXRA5)] were finally selected to establish a prognostic model, and their corresponding risk coefficients are shown in Figure 3C. According to the expression levels and regression coefficients, we calculated a risk score as follows:

$$\begin{array}{l} \mathrm{Risk}\;\mathrm{score}=\mathrm{expr}\left(\text{LGALS}1\right)\times \left(1.54e^{-1}\right)+\;expr\left(\text{DLL}3\right)\times \left(-4.82e^{-2}\right)\\ \;\;\;\;\;\;\;\;\;\;\;\;\;\;\;\;\;\;\;\;\;\;\;+expr\left(\text{ZFPM}2\right)\times \left(6.83e^{-2}\right)+expr\left(\text{LHX}6\right)\times \left(2.79e^{-2}\right)\\ \;\;\;\;\;\;\;\;\;\;\;\;\;\;\;\;\;\;\;\;\;\;\;+expr\left(\text{MXRA}5\right)\times \left(-1.11e^{-2}\right)+expr\left(\text{TMEM}56\right)\times \left(-1.18e^{-2}\right)\\ \;\;\;\;\;\;\;\;\;\;\;\;\;\;\;\;\;\;\;\;\;\;\;+expr\left(\text{CCL}23\right)\times \left(1.13e^{-2}\right)+expr\left(\text{FAM}155\text{B}\right)\times \left(3.08e^{-2}\right)\\ \;\;\;\;\;\;\;\;\;\;\;\;\;\;\;\;\;\;\;\;\;\;\;+expr\left(\text{ZSCAN}4\right)\times \left(1.19e^{-2}\right)+expr\left(\text{PCDHB}12\right)\times \left(-1.82e^{-2}\right)\\ \;\;\;\;\;\;\;\;\;\;\;\;\;\;\;\;\;\;\;\;\;\;\;+expr\left(\text{PRINS}\right)\times \left(-6.59e^{-2}\right)+expr\left(\text{FOXL}1\right)\times \left(-1.39e^{-3}\right)\\ \;\;\;\;\;\;\;\;\;\;\;\;\;\;\;\;\;\;\;\;\;\;\;+expr\left(\text{ASTN}1\right)\times \left(-6.39e^{-3}\right)+expr\left(\text{HMX}2\right)\times \left(6.38e^{-2}\right)\\ \;\;\;\;\;\;\;\;\;\;\;\;\;\;\;\;\;\;\;\;\;\;\;+expr\left(\text{HRASLS}\right)\times \left(4.28e^{-2}\right) \end{array}$$

**FIGURE 4:**
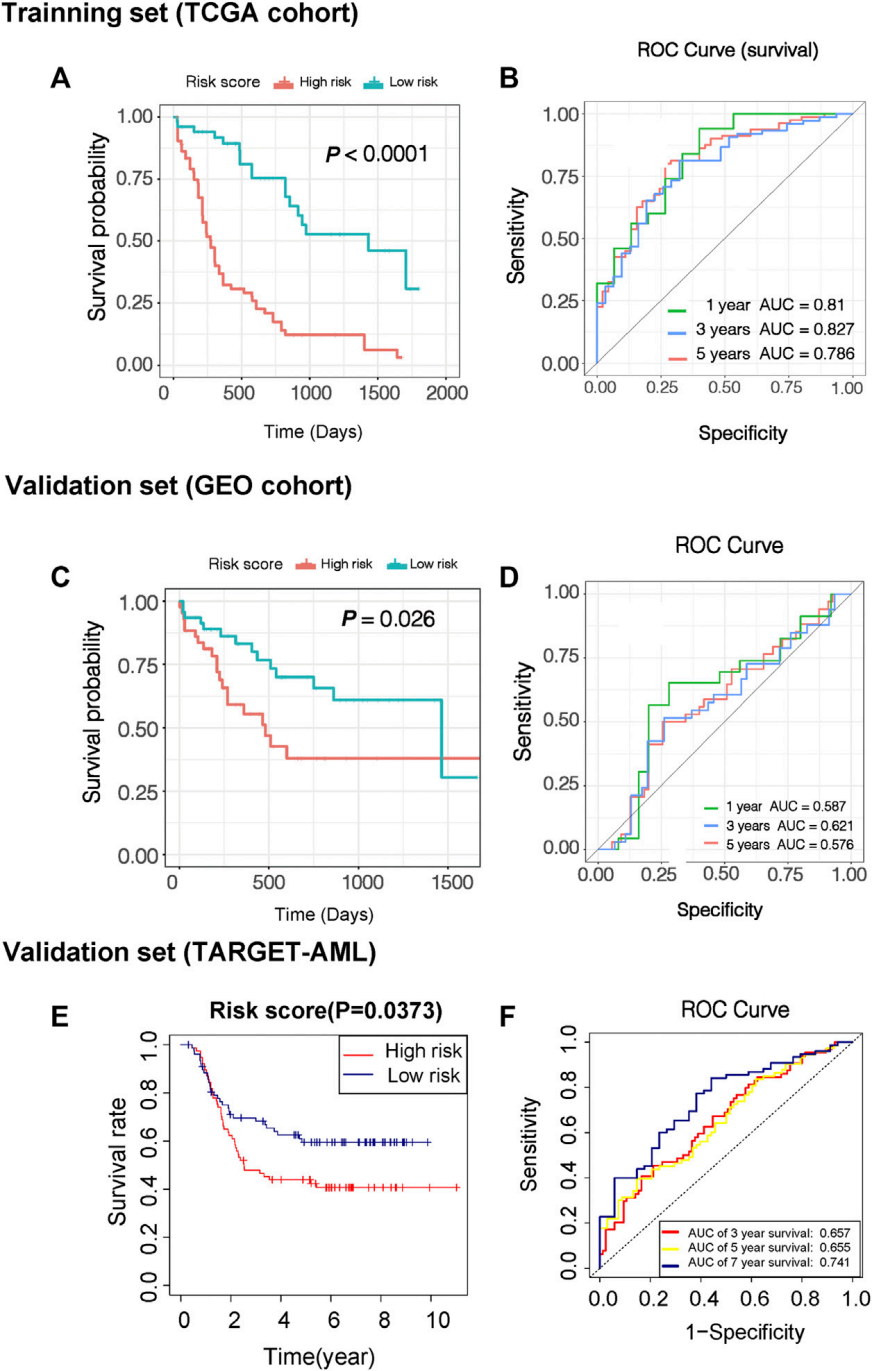
Evaluation of predictive efficacy of prognostic models. **(A)** KM survival curves of two risk score groups in the training set (TCGA-LAML data sets). **(B)** ROC curves in the training set (TCGA-LAML data sets), and the 3-year overall survival rates were 0.827. **(C)** KM survival curves of two risk score groups in the validation set (GSE71014). **(D)** ROC curves in the validation set (GSE71014) and the 3-year overall survival rates were 0.621. **(E)** KM survival curves of two risk score groups in the validation set in another validation set (TARGET-AML). **(F)** ROC curves in another validation set (TARGET-AML) and the 7-year overall survival rates were 0.741.

Based on the risk score, AML patients in the TCGA cohort can be grouped equally into low- and high-risk groups (Figure 3D). Patients in the low-risk group had less cell death and more survival time compared with those in the high-risk group (Figure 3E). The KM survival curves showed worse survival probability in the high-risk group than in the low-risk groups in both the training (Figure 4A) and validation sets (Figures 4C,E). In the training set, the area under the ROC curve (AUC) of the prognostic model for 1-, 3-, and 5-year survival time was 0.81, 0.827, and 0.786, respectively (Figure 4B). At the same time, the AUC of the prognostic model for the 3-year survival time was 0.621 in the GEO cohort (Figure 4D). Another validation from the TARGET database also performed good reproducibility, and the AUC values of 3- and 7-year survival times was 0.657 and 0.741, respectively (Figure 4F), indicating a good effect for predicting patients’ prognosis in both validation cohorts.

### Independent Prognostic Analysis of Risk Score and Pathological Features

**FIGURE 5:**
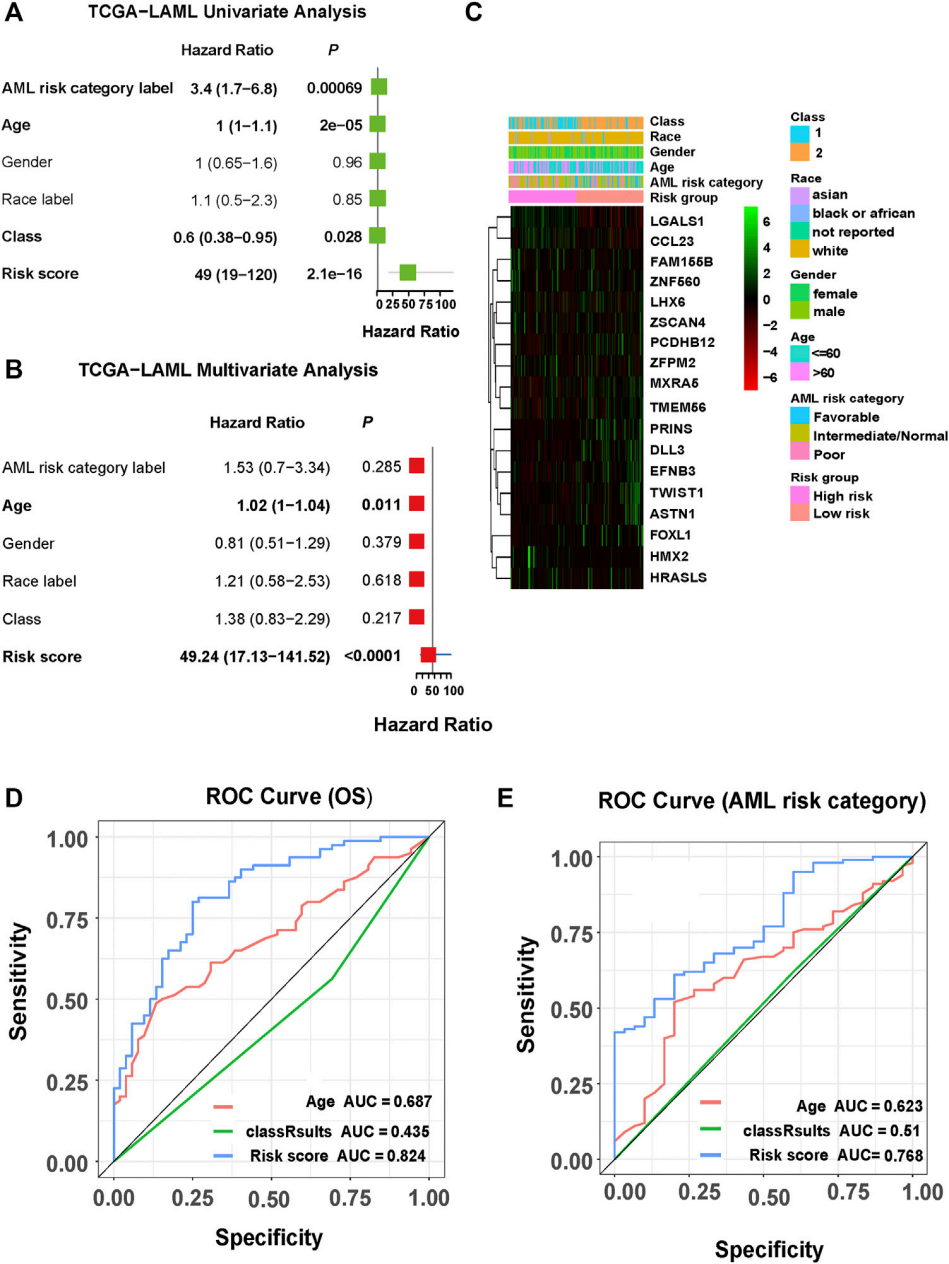
Correlations among clinicopathological characteristics, risk score, and prognostic value in TCGA cohort. **(A)** Univariate analysis of clinicopathological factors and risk score. **(B)** Multivariate analysis of clinicopathological factors and risk score. **(C)** Heat map for the correlations between the 18-gene risk signature and clinicopathological features. **(D)** ROC curve showing the prediction effect of risk score, age, and class 1/2 on survival rate. **(E)** ROC curve showing the predictive effects of risk score, age, and class1/2 on AML risk grouping.

**FIGURE 6:**
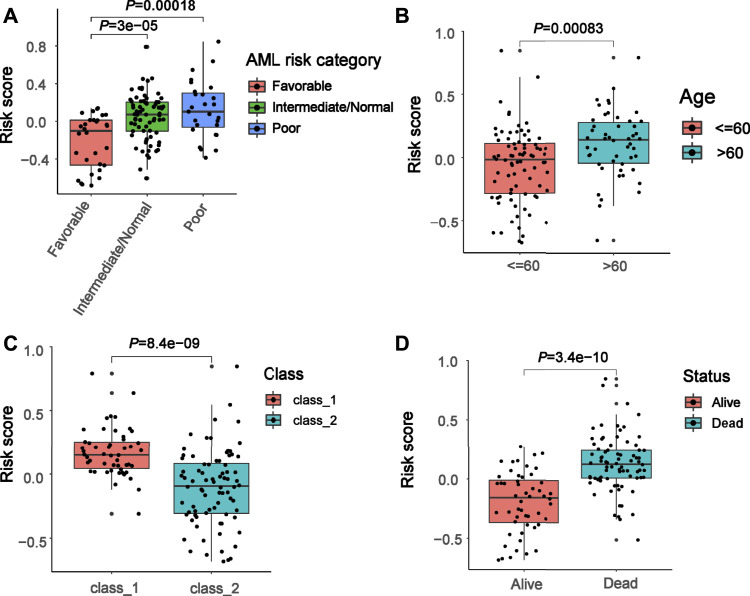
Association between risk score and clinical features. **(A–D)** Correlation of the risk score with AML risk category, age, class, and status.

To further explore the clinical value of the prognostic risk-scoring model, univariate and multivariate Cox regression analyses were performed on the TCGA cohort. In the univariate Cox analysis, AML risk category, age, class, and risk score were significantly associated with AML patient prognosis (*p* = 0.00069, *p* = 2 × 10^–5^, *p* = 0.028, *p* = 2.1 × 10^–16^, respectively, Figure 5A). Furthermore, the results of the multivariate Cox regression demonstrate that age and risk score are independent risk factors in AML patient prognosis (*p* = 0.011, *p* < 0.0001, respectively, Figure 5B). In addition, Figure 5C presents a heat map for the correlations between an 18-gene risk signature and clinicopathological features. Next, we performed an ROC analysis on these factors, and the results of the AUC value indicate that risk score has high accuracy to predict overall survival (OS) and AML risk category (AUC values = 0.824, AUC values = 0.768, respectively) compared with other factors (all AUC values < 0.7, Figures 5D,E). Moreover, we determined correlations between risk score and pathological features. We found that risk score was significantly associated with AML risk category, age, class, and status (all *p* < 0.05, Figures 6A–D).

### Establishment of Predictive Nomogram for AML Patients

**FIGURE 7:**
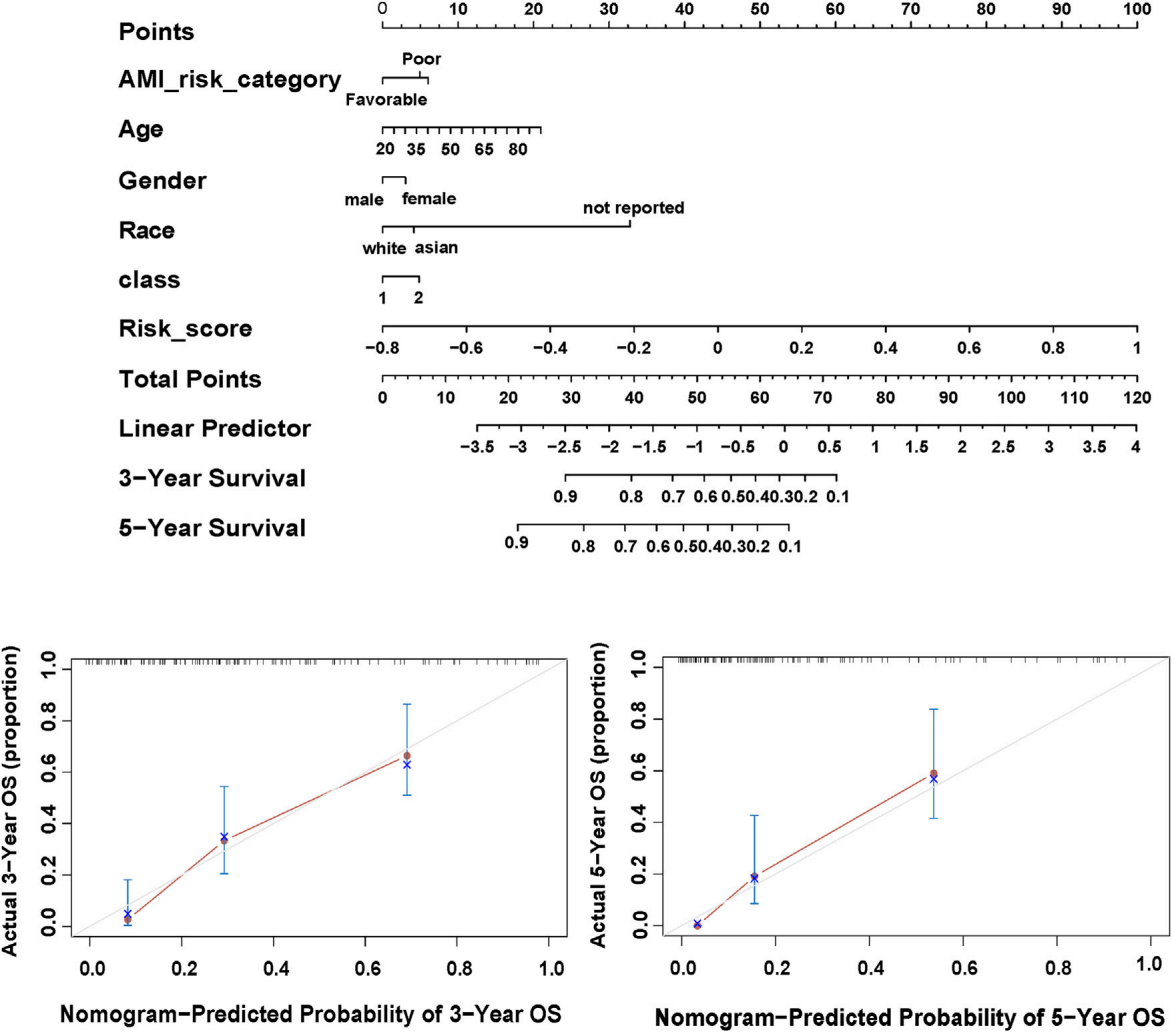
Nomogram of the TCGA-LAML. **(A)** Nomogram predicting 3- and 5-year OS for the AML patients. **(B,C)** Calibration curve for the nomogram.

According to the above regression analysis, we developed a nomogram containing our prognostic risk-scoring model and multiple clinical factors. In the TCGA cohort, AML risk category, age, gender, race, class, and risk score were eventually selected to establish an accurate predictive nomogram (Figure 7A). The calculated C index was to be 0.789, and the calibration plots of 3- and 5-year OS showed no deviations from the Platt calibration curves, indicating high predictive accuracy of nomogram (Figures 7B,C).

### Correlations Between the 18-Gene Signature and Immune Microenvironment

**FIGURE 8:**
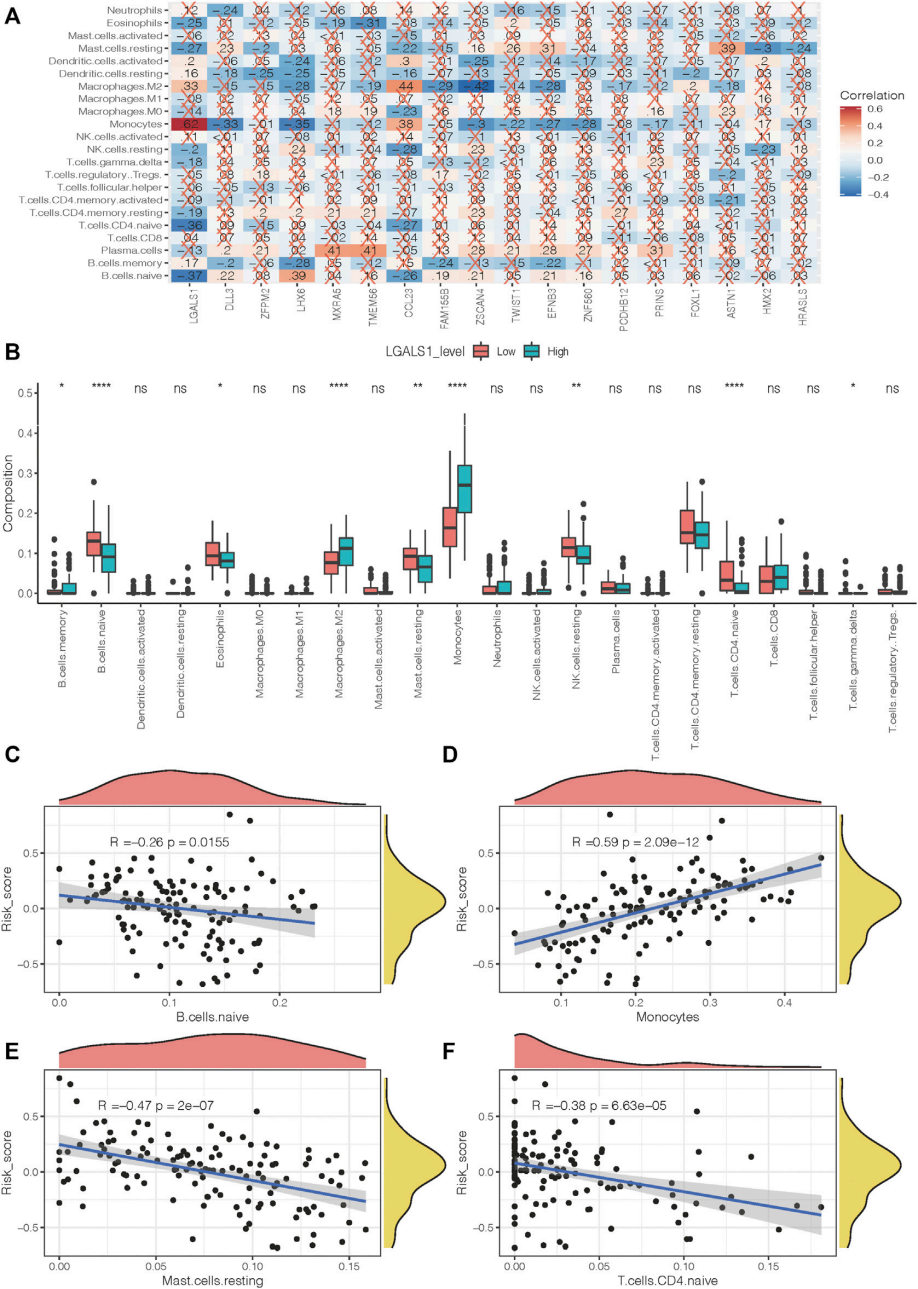
Immune infiltration level of prognostic signature. **(A)** Correlation between risk genes and different immune infiltration cell types. **(B)** The correlation of risk gene LGALS1 expression with immune infiltration level in the high- and low-risk groups. **(C–F)** The correlation of risk score with naïve B cells, monocytes, resting mast cells, and naïve T cells CD4.

To better investigate the interactions between the 18-gene signature and the immune microenvironment, we performed the CIBERSORT algorithm to evaluate the correlation of the prognostic risk-scoring model with tumor-infiltrating immune-cell fractions in AML patients. Figure 8A presents a heat map of the relationship between 18 risk genes and 22 immune infiltrating cells. Notably, LGALS1 shows the most significant association with immune activity. The LGALS1 level in the naïve B cells, eosinophils, resting mast cells, resting NK cells, naïve T cells CD4, and T cells gamma delta were more highly expressed in the low-risk groups compared with the high-risk groups, whereas LGALS1 level in B cell memory, macrophages M2, and monocytes were opposite (all *p* < 0.05, Figure 8B). Furthermore, we observed that a negative correlation of risk score with naïve B cells, resting mast cells, and naive T cells CD4 (R = −0.26, *p* = 0.0155; R = −0.47, *p* = 2 × 10^−7^; R = −0.38, *p* = 6.63 × 10^−5^, respectively, Figures 8C,E,F). With increasing risk score, the proportion of monocytes increase in a linear fashion (R = 0.59, *p* = 2.09 × 10^−12^, Figure 8D).

### Mutation Analysis of Prognostic Risk-Scoring Model

**FIGURE 9:**
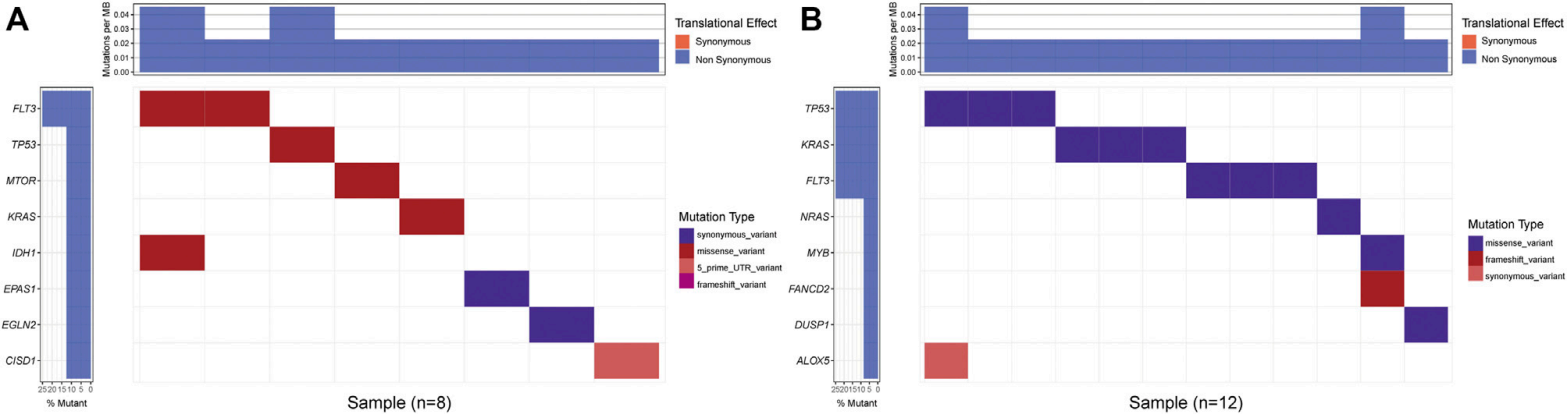
Mutation status of the prognostic model. **(A)** Low- and **(B)** High-risk groups mutation status.

We compared the mutation pattern between two risk groups, and a difference was found. The mutation frequency of the high-risk group was higher compared with the low-risk group at KRAS (25% vs. 12.5%), TP53 (25% vs. 12.5%). In addition, we found that mutation type was a missense variant in both risk groups (Figures 9A,B).

### Chemotherapeutic Drug Response of Prognostic Risk-Scoring Model

**FIGURE 10:**
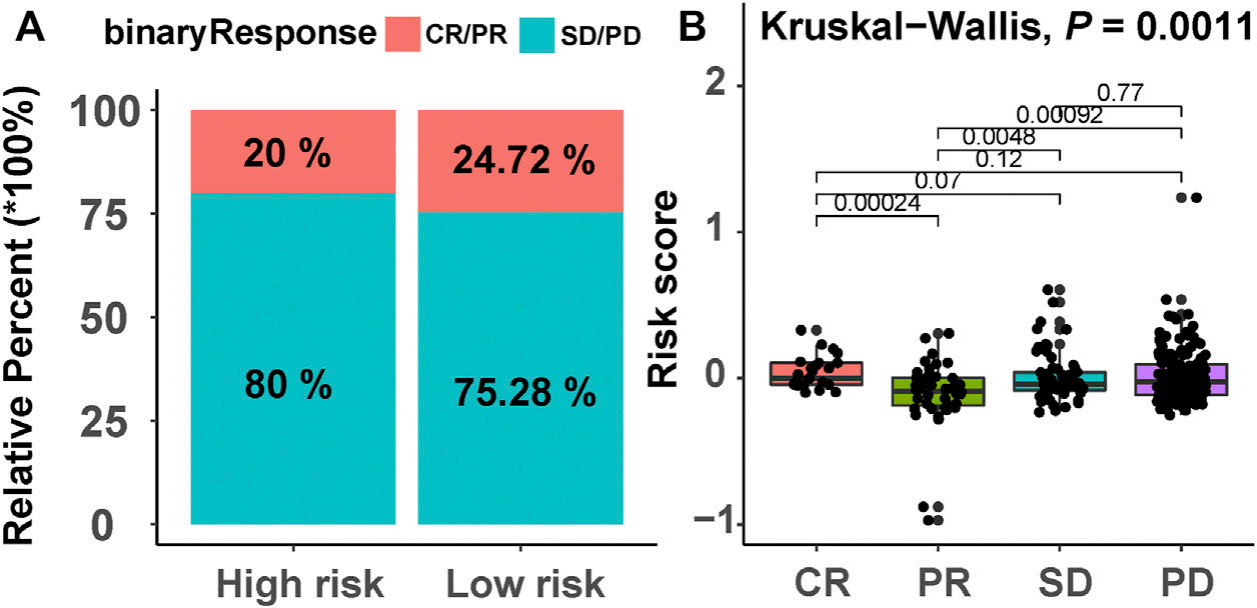
Chemotherapeutic drug response of the prognostic model. **(A)** The proportion of patients with different drug responses in the high- and low-risk groups. CR, complete response; PR, partial response; SD, stable disease; PD, progressive disease. **(B)** Distribution of patients with different drug response groups based on the risk score.

Due to the lack of drug information of AML patients, we selected a bladder cancer immunotherapy cohort (IMvigor210) to predict the efficacy of response to chemotherapy in the two risk groups. A higher chemotherapy response rate was observed in the low-risk group compared with the high-risk group (24.72% vs. 20%, Figure 10A). We also observed that there was a significant difference in the risk score distribution between the four groups of the patients with different responses to chemotherapy (Kruskal–Wallis, *p* = 0.0011, Figure 10B).

### Validation of Risk Genes

**FIGURE 11:**
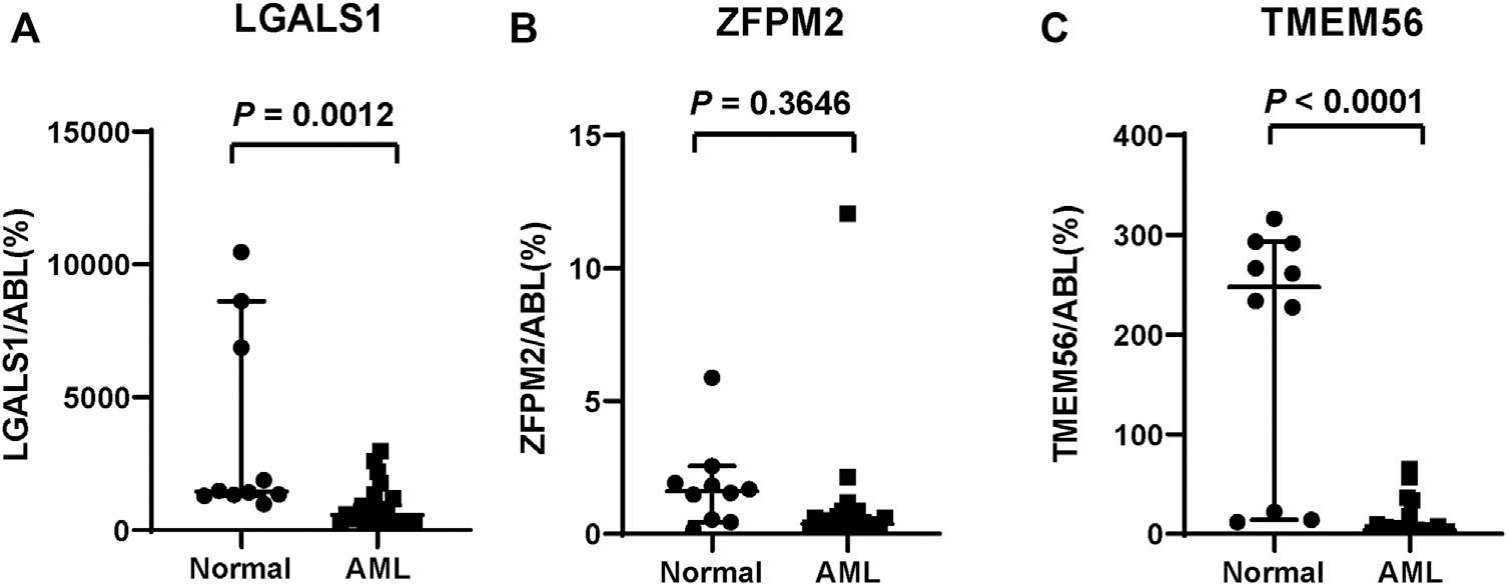
*In vitro* validation of three risk genes. **(A)** LGALS1, **(B)** ZFPM2, **(C)** TMEM56 mRNA expression level in the normal and AML samples.

We evaluated the expression of several risk genes in primary AML blasts by qPCR using 24 fresh or frozen *de novo* AML specimens, including the bone marrow and peripheral blood, and compared with that of 10 normal cases. The clinical characteristics and risk genes expression of these patients were presented in Supplementary Table S6. As shown in Figures 11A–C, ZFPM2 expression did not differ between the two groups (*p* = 0.3646), whereas LGALS1 and TMEM56 expression levels were significantly decreased in AML samples than that of the normal samples (all *p* < 0.05).

## Discussion

AML is the most common acute leukemia in adults with high mortality and unfavorable outcome. Among the traditional treatments, chemotherapy remains the major option for most AML cases even though a small number of patients may suffer from drug resistance and worse outcomes (Seo et al., 2010). Recently, mounting evidence demonstrates that ferroptosis can successfully eliminate resistant AML cells (Yu et al., 2015), which is proposed as a novel approach to killing malignant cells (Toyokuni et al., 2017). However, the extent to which ferroptosis influences patients’ chemoresistance and prognosis in AML requires further investigation. In this study, we identify genes that are affected by ferroptosis and develop a prognostic risk-scoring model to predict patients’ survival at the genetic level. The prognostic signature was employed to discriminate high- and low-risk patients, and high-risk patients had a worse outcome and worse responses to chemotherapeutic drugs.

Current studies identify a ferroptosis- or autophagy-related long noncoding RNA signature for predicting prognosis in the patients with AML (Li R. et al., 2021; Zhao et al., 2021; Zheng et al., 2021). Immune- or immune checkpoint–related gene signatures were also developed and validated in AML patients (Li R. et al., 2021; Jiang et al., 2021). However, the results of these studies were biased because they mainly focus on a small number of genes that were related to special functions instead of the whole gene population. Our study grouped AML patients into two clusters based on 261 ferroptosis-related genes, and candidate genes were screened between two clusters. These genes were not only associated with ferroptosis, but also presented a comprehensive analysis of the whole gene population of AML patients. In addition, these studies do not establish the prognostic nomogram and lacked further investigations for the risk signature. Our study not only developed an effective nomogram incorporating both prognostic signature and clinical elements, but also analyzed the correlations between risk signature and immune activity and response to chemotherapy.

Our prognostic model involves 18 genes affected by ferroptosis. According to the risk value of each gene, ZFPM2, ZNF560, ZSCAN4, HMX2, HRASLS, LGALS1, LHX6, CCL23, and FAM155B were regarded as risk genes related to poor prognosis in patients with AML, whereas MXRA5, PCDHB12, PRINS, TMEM56, TWIST1, ASTN1, DLL3, EFNB3, and FOXL1 were associated with favorable prognosis. Among all these genes, higher expression of TWIST1 (Wang et al., 2015) and DLL3 (Takam Kamga et al., 2019) is revealed to be correlated with favorable prognosis in the patients with AML, whereas higher expression of LGALS1 (Ruvolo et al., 2020) has a poor outcome, which is consistent with our prediction. As a critical transcription factor involved in epithelial-mesenchymal transition, TWIST1 overexpression can enhance the susceptibility to chemotherapy drugs by promoting cell cycle entry and, thus, improve AML patients’ outcomes (Chen et al., 2015; Wang et al., 2015). DLL3 was an atypical Notch ligand that has been investigated in many tumors as a therapeutic target (Matsuo et al., 2021). In AML, improved survival was observed in high expression of DLL3, and it may function by cell proliferation regulation (Yan et al., 2010; Takam Kamga et al., 2019). LGALS1 mainly participated in inducing tolerogenic programs and contributed to tumor cell immune evasion (Cagnoni et al., 2021). Generally, LGALS1 exerted a tumor-promoting effect by blocking tumor suppressors such as p53 and promoting drug resistance in AML (Ruvolo et al., 2020; Li X. et al., 2021). However, the expression and clinical outcome of 18 genes in the patients with AML are still ambiguous. Herein, we selected LGALS1 and other two risk genes (ZFPM2, TMEM56) that have not been reported previously in AML to validate their expression level in the clinical samples. Our PCR experiments show that LGALS1 and TMEM56 expression had significant differences between the normal and AML samples. However, LGALS1 was underexpressed in AML and the expression of LGALS1 was quite different from the previous research (Ruvolo et al., 2020). Various factors, such as patient-to-patient variation, could have contributed to the discrepancy. Therefore, we ideally need more data to evaluate this issue. Additionally, RNA sequencing data of these AML patients is also required to collect for verifying the risk-scoring model, which is better to integrate the gene expression from the TCGA, GEO, etc., data sets with the measurements.

As expected, the 18-gene signature can well predict AML patients’ prognosis compared with the traditional AML risk category. AML patients can be divided into high- and low-risk groups according to risk score, and we found that the survival was significantly different between the two risk groups. In ROC analysis, the AUC values of 1- and 3-year survival were greater than 0.8 in the patients with AML from the TCGA cohort, indicating a superior predictive power in comparison with Zhao et al. (2021) (3-year AUC = 0.706) and Jiang et al. (2021) (3-year AUC = 0.711). Notably, risk score was identified as the independent prognostic factor after univariate and multivariate analyses. Among all the clinical factors, the risk score most significantly affected the AML risk category and survival of AML patients, which can effectively guide prognostic prediction. Importantly, we successfully constructed a prognostic nomogram that combined the risk signature with clinical parameters and extend the clinical applicability of our prognostic model.

At present, evading antitumor immune responses is considered to be an important cause of progression or relapse of AML (Taghiloo and Asgarian-Omran, 2021), and thus, immunotherapy has been widely investigated in the clinical treatment of AML. In the immune evasion mechanisms, multiple immune cells are involved. For example, AML blasts may block the effector functions of T and NK cells, increase immunosuppressive cells such as macrophages M2 and monocytes and decrease immunoreactive cells such as naive B cells and resting mast cells (Tettamanti et al., 2021). In our study, we found that LGALS1 was most closely related to immune infiltration cells among these 18 genes. As key regulators of tumor immune evasion, high LGALS1 expression in AML patients was associated with higher macrophages M2, monocytes infiltration, and lower naive T cells CD4, naive B cells, and resting mast cells infiltration, which remained consistent with a previous study (Tribulatti et al., 2012). Interestingly, our study also indicates that there is a certain correlation between risk score and immune cell infiltration. The risk score was negatively correlated with naïve B cells, naive CD4^+^ T cells, and resting mast cells, whereas it is positively related to monocytes. Naïve B cells can differentiate into antibody-secreting plasma cells when they encounter a new antigen (Siegrist and Aspinall, 2009). Naive CD4^+^ T cells can also differentiate into various subsets to attain specialized effector functions, which play a vital role in tumor immunity (Huang et al., 2012). Resting mast cells are immunoreactive cells that are related to better survival (de Alencar et al., 2020). In opposition, monocytes play an important role in tumor growth and progression (Shao et al., 2018). Therefore, a negative correlation of risk score with naïve B cells, naive CD4^+^ T cells, and resting mast cells and the positive correlation with monocytes suggests that the 18-gene signature is tightly associated with immune-active status in the tumor microenvironment. However, more in-depth research combining clinical samples is needed to clarify this relationship between risk score and the immune microenvironment of AML.

Gene mutation is another important cause of tumorigenesis and drug resistance. In our study, we found that the high-risk patients had higher KRAS mutations compared with the low-risk patients. Previous studies demonstrate that clonal mutations in KRAS are related to therapy resistance (Jerchel et al., 2018). Therefore, poor survival of high-risk patients may also be associated with KRAS mutation, which can cause chemoresistance.

Finally, we explored the effectiveness of risk score in predicting chemotherapy response, and the results demonstrate that the chemotherapy response rate of high-risk patients was lower than that of low-risk patients. These results suggest that our model could predict chemotherapy response to a certain extent.

There are also some limitations in this study. First, although we provide a nomogram for predicting survival in AML, more prospective studies are needed to confirm the reliability of this nomogram. Second, the data preprocessing and the background of patients of three cohorts (TCGA-LAML, GSE71014, TARGET-AML) were different, resulting in the different cutoff values and biased prediction efficacy. Third, there was also a paucity of drug data on chemotherapy in the patients with AML, and more validation data sets are required to confirm the applicability of our prognostic model. Finally, we still need more experimental evidence to prove our conclusion and elucidate the exact mechanism of these 18 genes in AML progression, immune therapy, and drug resistance.

## Conclusion

In our study, we construct a novel prognostic signature for efficiently predicting AML patients’ prognosis based on ferroptosis-related cluster. We further found that high-risk patients of AML had worse survival and reduced response to chemotherapy, which may provide therapeutic options for AML patients.

## Data Availability

Publicly available data sets were analyzed in this study. This data can be found here: Publicly available data sets can be obtained from the TCGA (https://portal.gdc.cancer.gov/), GEO (https://www.ncbi.nlm.nih.gov/gds/), and TARGET databases (https://ocg.cancer.gov/programs/target).

## Supplementary Material

Supplementary Figure 1 Functional enrichment analyses of the DEGs between two subtypes. **(A)** Enrichment results of GO molecular function containing Ferroptosis-related genes. **(B)** Enrichment results of GO biological processes containing Ferroptosis-related genes. **(C)** Enrichment results of GO cellular components containing Ferroptosis-related genes. **(D)** Enrichment results of KEGG pathway.

Supplementary Figure 2 Top 3 prognostic DEGs with the most significant *p*-value. **(A–C)** Gene expression (ITGAX, LGALS1, MIR551A) between the two subtypes and KM survival curves based on the high and low expression of them. ****represents *p* < 0.0001.

Supplementary Table 1 Analysis results of GO BP.

Supplementary Table 2 Analysis results of GO MF.

Supplementary Table 3 Analysis results of GO CC.

Supplementary Table 4 Analysis results of KEGG.

Supplementary Table 5 Analysis results of univariate Cox regression.

Supplementary Table 6 Patient characteristics and risk genes expression of primary AML patient samples. AML, acute myeloid leukemia; F, female; M, male; BM, bone marrow; PB, peripheral blood; WBC, white blood cells; CR, complete response; EFS, event free survival; OS, overall survival.
