# Supplementary figures and images for: Identification and Validation of a Prognostic Risk-Scoring Model Based on Ferroptosis-Associated Cluster in Acute Myeloid Leukemia
Source: Front Cell Dev Biol. 2022 Jan 21;9:800267. doi: 10.3389/fcell.2021.800267 (PMC8814441; doi:10.3389/fcell.2021.800267)

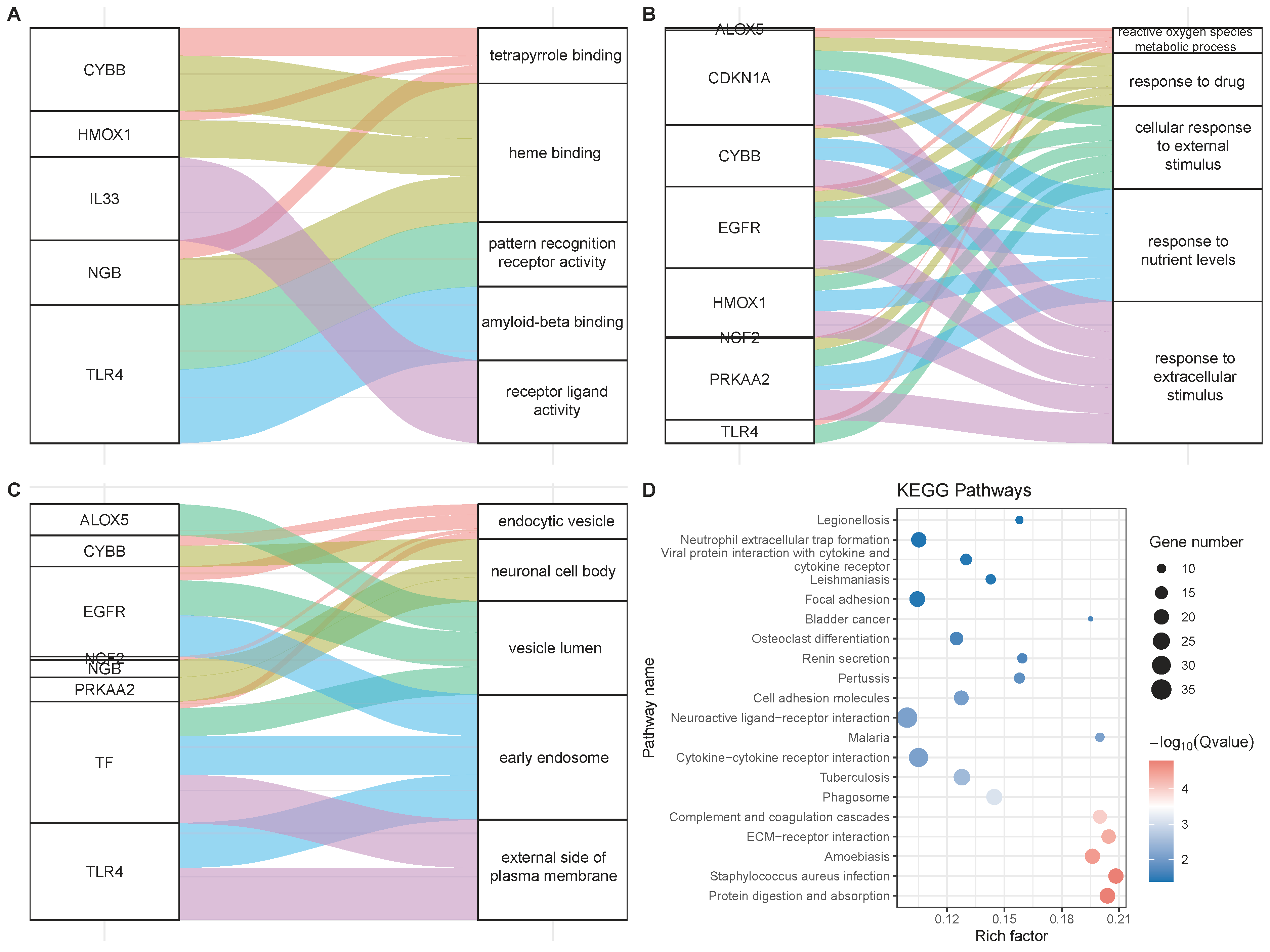

Supplement: Supplementary file 1 [file Image1.TIFF]

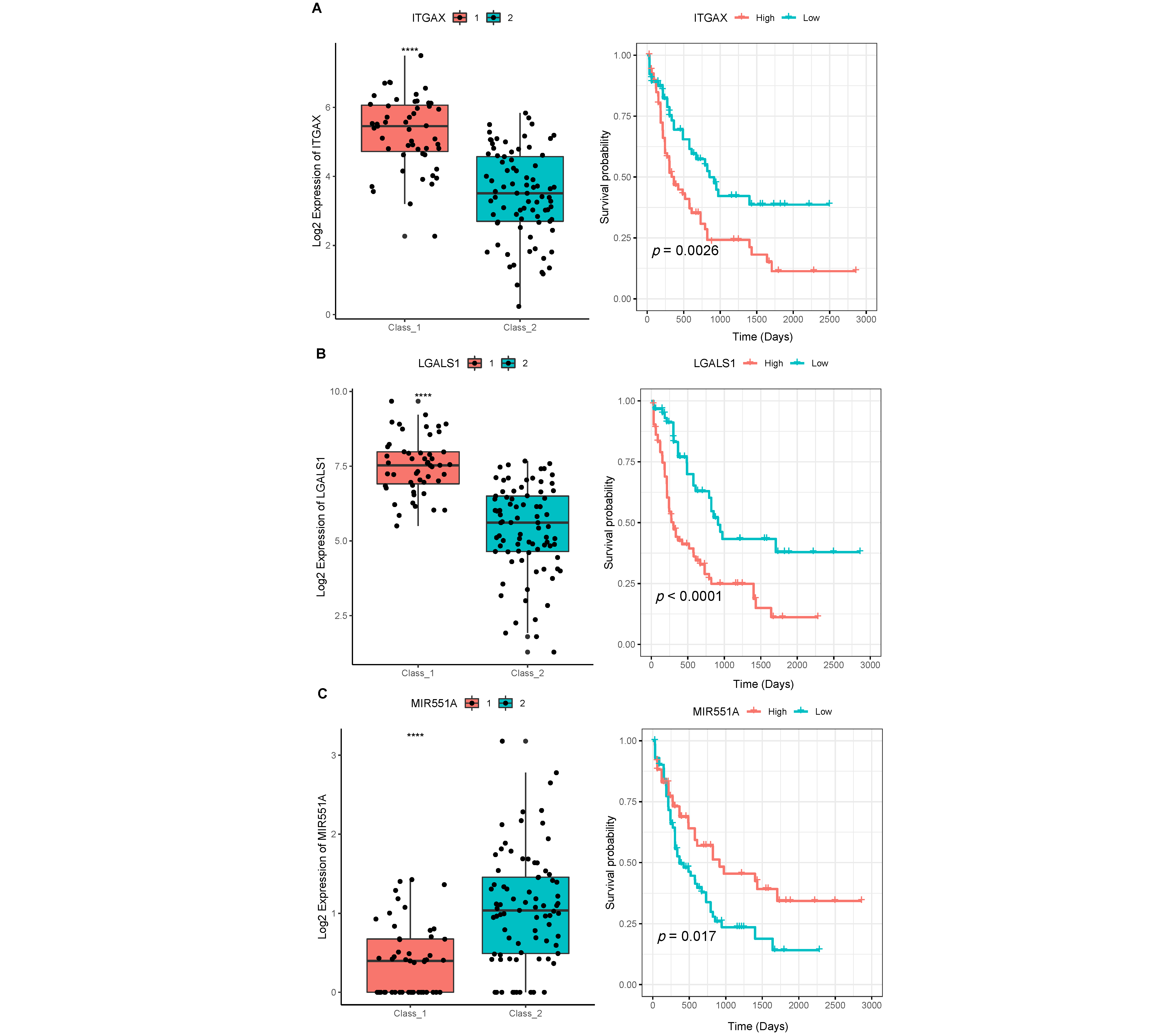

Supplement: Supplementary file 6 [file Image2.TIFF]
